# Prescribing Antibiotics in Rural China: The Influence of Capital on Clinical Realities

**DOI:** 10.3389/fsoc.2020.00066

**Published:** 2020-09-04

**Authors:** Meixuan Chen, Paul Kadetz, Christie Cabral, Helen Lambert

**Affiliations:** ^1^Population Health Sciences, Bristol Medical School, University of Bristol, Bristol, United Kingdom; ^2^Department of Anthropology, Durham University, Durham, United Kingdom; ^3^Center for Global Health, School of Public Health, Zhejiang University, Hangzhou, China

**Keywords:** antibiotic resistance, AMR, clinical practice, rural China, cultural capital, social capital, economic capital

## Abstract

Primary care clinicians in rural China are required to balance their immediate duty of care to their patients with patient expectations for antibiotics, financial pressures, and their wider responsibilities to public health. The clinicians in our sample appear to make greater efforts in managing immediate clinical risks and personal reputation than in considering the long-term consequences of their actions as potentially contributing to antimicrobial resistance. This paper employs Bourdieu's theory of capital to examine the perspectives and practices of Chinese primary care clinicians prescribing antibiotics at low-level health facilities in rural Anhui province, China. We examine the institutional context and clinical realities of these rural health facilities and identify how these influence the way clinicians utilize antibiotics in the management of common upper respiratory tract infections. Confronted with various official regulations and institutional pressures to generate revenues, informants' desire to maintain good relations with patients coupled with their concerns for patient safety result in tensions between their professional knowledge of “rational” antibiotic use and their actual prescribing practices. Informants often deferred responsibility for antimicrobial stewardship to the government or upper echelons of the healthcare system and drew on the powerful public discourse of “*suzhi*” (human quality) to legitimize their liberal prescribing of antibiotics in an imagined socioeconomic hierarchy. The demands of both practitioners' and patients' social, cultural, and economic forms of capital help to explain patterns of antibiotic prescribing in rural Chinese health facilities.

## Introduction

Pathogenic resistance to antimicrobials (AMR) is widely acknowledged as a key global health challenge (World Health Organization, 2012, 2015; United Nations, 2016). Antibiotic resistance poses a particularly severe threat in China, a leading consumer of antibiotics in humans, agriculture, and livestock, with high rates of antibiotic resistance, and in 2018, it was projected to have the highest mortality rates in the world from AMR by 2050 (The Center for Disease Dynamics Economics Policy, 2018). In order to mitigate this threat, it is imperative to understand all the factors influencing the development of antibiotic resistance in China at this time.

Since antimicrobial resistance begins to evolve as soon as new antibiotics begin to be used, the push to develop new antimicrobials is not likely to resolve AMR. Many drivers of AMR are anthropogenic, and as Smith (2015, p. 1–2) argues: “The conditions promoting the biological mechanisms of antimicrobial resistance are deeply social, shaped by cultural, political, and economic processes. Although the mechanism for antimicrobial resistance is biological, adherence to antimicrobial stewardship is fundamentally social [and] as a social challenge it demands social solution[s].” Thus, if AMR is ultimately an outcome of clinical praxis, as well as embodied dispositions influenced by the social, cultural, and economic capital forming the habitus of particular groups^1^, then studies that foster an understanding of these sociocultural drivers could potentially identify and guide the changes needed to effectively manage AMR.

Individual practices are structured by the medical and broader social systems in which these individuals work, while their practices reciprocally shape these systems. According to Bourdieu (1986), “It is in fact impossible to account for the structure and functioning of the social world unless one reintroduces capital.” Stated simply, forms of capital accumulate and distinguish one's place and one's assigned value in a given social hierarchy according to what one knows, who one knows, and what one owns.

Bourdieu's theory of capital has been used to analyze a range of healthcare issues including health inequalities (Pinxten and Lievens, 2014), health achievement (Meinert, 2004), healthcare provider choice (Collyer et al., 2015), maternal practices (McKeever and Miller, 2004), medical elites (McDonald, 2014), and medical education (Brosnan, 2011). Broom et al. (2014, 2017, 2018, 2020), Broom and Doron (2020) have specifically used Bourdieu's framing of the role and production of social capital and habitus to unpack the complex antibiotic prescribing behaviors of physicians. They argue that “antibiotic decisions are relational and negotiated, and tied to patient expectations as well as the lay-expert dynamics that influence these” (Broom et al., 2014, p. 82). They conclude that antibiotic prescribing is particular to the acquisition of social capital through the habitus of a given context, regardless of best practices or therapeutic guidelines.

To better understand the complex relationships between the myriad social drivers influencing human antibiotic use in rural China, we build on and expand Broom's use of Bourdieu to this work by unpacking the prescribing behaviors of clinicians in six rural health facilities—three village clinics (VCs) and three township health centers (THC)—in three counties of Anhui province, China. We interpret the use of antibiotics by VC and THC doctors for patients presenting with symptoms of upper respiratory tract infections (RTIs) as an outcome of their negotiations of cultural, social, and economic capital^2^. We examine both doctors' and patients' social, economic, and institutional positioning in China's health systems and their influence on antibiotic prescribing.

## Methods and Settings

The findings presented here draw on direct observation, semi-structured interviews, and informal conversations with a sample of village doctors and township level physicians in three counties of Anhui Province, China (referred to here as Sites 1, 2, and 3), between January 2017 and June 2018. This qualitative research was part of a wider interdisciplinary study investigating pathways to antibiotic use and antibiotic resistance in clinical and community settings in rural Anhui province, details of which are reported elsewhere (Zhao et al., 2019). In each county, one THC and one VC were selected for patient recruitment and direct observation of the clinical encounter.

Like most rural primary healthcare units in China, both THCs and VCs provide walk-in clinics for outpatients with no appointment system or triage, although THCs also have inpatient wards. THCs are the healthcare units at the bottom of the state health system hierarchy and are literally referred to in Chinese as the “Hygiene Hall” (*Weisheng Yuan*, 卫生院). After the SARS epidemic in 2003, THCs were officially designated to manage public health in rural areas by incorporating local VCs into the healthcare system. VCs are subcontracted to carry out public health functions, such as vaccination on behalf of the THC, which the THC manages, in addition to overseeing the public health activities and records of VCs. There is a ratio of between 3 and 6 VCs per THC^3^.

The three VCs studied are diverse in terms of their staff numbers, division of labor, and their use of electronic and paper information systems. For example, three doctors work in Site 1 VC. All three provide patient consultations and use electronic patient records. By contrast, site 2 VC has four staff members whose roles differ and include a senior doctor, who is officially retired but has returned to work and undertakes most patient consultations; his younger brother, who is the VC director and cashier and who also inputs the electronic patient records; a third staff member who acts as the in-house pharmacist; and a fourth doctor who mainly undertakes home visits. Patient volume and staffing likewise vary across the three THCs studied. Two serve as the central health facilities for the local town (*zhen zhongxin yiyuan*, 镇中心医院) and receive a large number of outpatients every year. Site 1 THC has two outpatient doctors, one of whom is trained in traditional Chinese medicine (TCM), while the other is biomedically trained. Site 2 THC employs nine doctors in total, of which seven doctors staff inpatient wards and outpatient clinics in rotation. Site 3 THC is smaller and quieter than the other two, with three doctors available for outpatient consultations.

Unlike the predominantly biomedically trained physicians who work in THCs, VCs are overseen by so-called village doctors (*cunyi*, 村医), a carry-over of the minimally trained community health workers formerly known as barefoot doctors (*chijiao yisheng*, 赤脚医生) during the Maoist period. Barefoot doctors were often the only health workers in the local level brigade health units that were dissolved under the economic reforms of Deng Xiaoping in the 1980's (Hu et al., 2017). Although, since the 1990's, village doctors have been required to complete a minimum of 2–3 years of specialist medical education in order to practice, they have not passed the examination to obtain a license to practice as an assistant physician and therefore do not have the same social standing as trained physicians (*Ibid*.)^4^.

### Data Collection and Analysis

From the six healthcare facilities participating in this study, a total of 21 village doctors and township physicians were observed during their daily consultations with patients, all but 2 of whom were followed up for semi-structured interviews toward the end of the 4th month of observation in each site. A topic guide was drafted for the interviews, which was then piloted in Site 1 and revised for Sites 2 and 3. To protect confidentiality, each informant interview was anonymized via a study ID. Verbal informed consent was obtained before clinical observation and at the beginning of each interview.

All VC informants held the Certificate of Village Practitioner, issued by the local County Health Commission. Thirteen informants had received training in a 2 or 3-year vocational health college (*weixiao*, 卫校), of which nine were trained solely in biomedicine (*linchuang zhenduan*, 临床诊断), three were trained in traditional Chinese medicine (*zhong yi*, 中医), and one informant had received both biomedical and TCM training (1-1-20170704). Three village doctors reported that they had no formal health training but had apprenticed with family members or been taught via correspondence courses (2-2-20180124). Table 1 provides a summary of the demographics of the sample.

Table 1Summary of sample demographics.

**Table:** 

**Dataset**	**Site 1**		**Site 2**		**Site 3**		**Total**	
Clinician Interviewed	4		11		4		19	
	VC	THC	VC	THC	VC	THC	VC	THC
	2	2	3	8	1	3	6	13
Sex								
**Female**			**Male**				**Total**	
2			17				19

**Table:** 

Number of Years in Practice				
10–15 years	15–20 years	20–30 years	30–40 years	Over 40 years
2	2	11	1	3
Formal medical training in clinical medicine (临床医学专业): 2–3 years in Secondary Vocational Health College (literally Hygiene College, *Zhongzhuan* or *Dazhuan Weixiao* 中专大专卫校)				
**Secondary vocational medical school**			**No formal medical training**	**Unknown**
13			3	3
Specializes in biomedicine (*xiyi*)	Specializes in TCM (*zhongyi*)	Specializes in both biomedicine and TCM		
10	4	1		

The majority of interviews were audio-recorded and transcribed by the research team. Anonymized transcripts and observation notes were stored and coded in the software package NVivo 11 (4 from site 1; 13 from site 2; 4 from site 3). Thematic analysis, as described by Charmaz (2006) and Boyatzis (1998), was performed. Themes were also analyzed according to a theoretical framework employing Bourdieu's concepts of social, cultural, and economic capital. A list of coding labels was developed through a process of inductive coding. Ethical approval was obtained from the Anhui Medical University Research Ethics Committee.

## Results: Uncertainty and Risk in Antibiotic Prescribing

This study reveals multiple factors that influenced antibiotic prescribing in our sample of medical practitioners. Several informants discussed clinical uncertainty (*bu queding*, 不确定) as a primary reason for prescribing antibiotics. Clinical uncertainty was frequently mentioned as a common issue that clinicians face in rural Chinese primary care settings when deciding upon treatments. This uncertainty is an outcome of two challenges: correct differential diagnosis of the overall category of infection and the ability to distinguish the specific type of pathogen present.

A majority of informants described using broad treatments due to the difficulty in differentiating between bacterial and viral infections. One informant noted:

As a clinician, I think that if you aren't sure, then all you can do is choose to use both types [of medicine] together. Because if you can't confirm what [the pathogen] is, and if you only use, say, antibiotics (*kangsheng su*, 抗生素), [that is] anti-inflammatories (*xiaoyan yao*, 消炎药), when in fact it is a viral infection (*bingdu ganran*, 病毒感染), then you won't have good results. So all you can do is use the two together in combination (1-2-4).

Here, the clinician refers to “antibiotics” (*kangsheng su)* and “anti-inflammatories” (*xiaoyan yao*) as the same thing, in addition to being medicines that do not work for viral infection. Informants commonly claimed that when they were unable to make a definitive diagnosis of bacterial or viral infection; they would need to prescribe both antibiotics *and* antivirals, a practice that was frequently observed in the clinics. They rationalized that, thereby, they could be certain that at least one of these two types of medicines would work for patients. Informants attributed the difficulty of a precise diagnosis to a lack of laboratory support in rural primary care settings. Thus, when unable to differentiate between the type of pathogen causing an infection, the practice of prescribing both antiviral and antibacterial medicines was widespread and regarded as normal, despite informants' knowledge that antibiotics are for use in bacterial infections and are ineffective for viral infections, which often include upper respiratory tract infections.

A second type of clinical uncertainty demonstrated by informants was in distinguishing the exact type of bacteria causing infection. Township clinic informants reported, and were frequently observed, using blood tests and chest x-rays as proxy diagnostic tools. However, since only microbiological cultures, which are not available on site, can identify the specific group of bacteria causing infection, informants pointed out that they are unable to determine accurately whether to prescribe broad-spectrum or narrow-spectrum antibiotics. As one THC informant noted:

If you aren't certain which type of microbial infection (*xijun ganran*, 细菌感染) it is, then it is generally agreed that you should use broad-spectrum antibiotics (*guangpu kangsheng su*, 广谱抗生素); sometimes combining two or three is definitely a bit better. Why do I say a bit better? Because you aren't certain which type of antibiotics (*kangsheng su*) is better for the infection in question (2-1-20180201).

Faced with such inexact methods of identification, several informants spoke candidly that in such cases they would prescribe at least two broad-spectrum antibiotics, such as amoxicillin or cephalosporin (*toubao*, 头孢), along with another type of antibiotic. Informants revealed a rationale that two broad-spectrum antibiotics would deal with more than one group of bacteria, or bacteria located at two or more locations in the body, such as the throat and chest. Another informant (2-1-20180201) described this therapeutic goal as encircling the pathogenic agent, or literally “to form a large enclosure around the pathogen,” whereby several types of antimicrobials are combined in order to broadly treat any infection, regardless of the actual pathogenic agent. Several informants reported to employ such strategies to derive a sense of certainty that they were making efforts to fight all probable causes of infection. This pattern of prescribing is officially labeled “combined use of medicine” (*lianhe yongyao*, 联合用药) and is perceived by informants to more reliably treat all possible causative pathogens, despite the fact that the use of “combination medicine” is widely associated with the development of AMR and has been explicitly banned by official guidelines in recent years specifically for this reason (National Health Commission of the People's Republic of China, 2015, Document No. 43).

However, clinical uncertainty was not the only driver of frequent antibiotic prescribing in our sample. Several informants discussed the benefits of using antibiotics to reduce risk, both to the patient and to their professional reputation. One informant considered it justifiable to prescribe antibiotics in some cases in order to avoid “medical risk” (*yiliao fengxian*, 医疗风险), in situations where “antibiotics can be used” (*[kangsheng su] keyong bukeyong*, “[抗生素] 可用可不用”'), such as “when the patient has a viral infection, and [the patient] is in good physical condition or has good immunity. Then in the first two days, it is OK not to use antibiotics” (2-1-20180115). This informant estimates that the number of cases where there is “no necessity for antibiotics” accounts for 20–30% of the THC antibiotic prescriptions. Yet, he felt that “even if antibiotics are used for 10% [of these cases], it cannot be regarded as [drug] abuse (*lanyong*,滥用), as medical risk is present. The Health Ministry told us not to use antibiotics.” However, the frontline clinicians, depending on the extent of their medical expertise, would often use antibiotics in order to “prevent risk” [to both clinicians' reputations and patient's health] (*fangfan fengxian*, 防范风险).

If [the] clinician's medical capability is poor and they've got no guts, then they use it. As long as there are no allergies to the antibiotic, we use it. Every [doctor] is using it anyway (2-1-20180115).

Interestingly, this informant is deeply critical of what he sees as “antibiotic abuse” by his colleagues at the health service unit where he works. He stressed this occurs at the “lowest level” (*diceng*, 底层) primary care unit and implies that these clinicians lack the medical skills and courage to treat patients effectively, without relying on antibiotics. He and other informants suggest that “frontline clinicians,” such as his colleagues, may use antibiotics to minimize the potential risks they perceive; of being accused by patients for having failed to prescribe antibiotics in case of serious infection. Two other informants (2-1-20180201; 2-1-20180131) similarly mentioned that they couldn't risk putting their patients' well-being in any immediate danger, as their first reason for prescribing antibiotics.

The perception of using antibiotics for risk reduction also provides a rationale for their prophylactic use but creates conflicts for doctors in demonstrating appropriate practice. One THC informant commented:

Some doctors have to think about safety, right? This is preventive use, because I don't know yet if there is new infection, so I use a little bit of antibiotics first (2-1-20180201).

This informant perceives that he is demonstrating his concern for patient safety with his prophylactic use of antibiotics. By stressing that he would use only “a little bit” of antibiotics, however, he also attempts to demonstrate his awareness of the policy limiting antibiotic use, while simultaneously diminishing the clinical significance of his practice as having an impact on antibiotic resistance.

Greater clinical risk is associated with the perceived vulnerability of some patients, particularly of the elderly and children. When patients' symptoms initially indicate viral infection at the time of consultation, informants report being concerned that the viral infection may develop into a bacterial infection with serious complications, such as pneumonia. One doctor reported that he “would rather” use antibiotics prophylactically for those whom he perceives to be more vulnerable: “A child's situation can change rapidly in a short period of time” (2-1-20180131).

Another circumstance that prompts clinicians to prescribe antibiotics for “preventive use” is for night-time emergencies at THCs, when neither blood tests nor x-rays are available to support a diagnosis. One THC doctor states:

We might use some [antibiotic] when it's not clear whether something is a viral or bacterial infection, because you can't do a blood test in the evening or especially in emergency situations where there isn't the time. At night you don't actually know what the cause is. Perhaps we may as well use antibiotics (*kangsheng su*), but the ones we use are usually not the type that require a skin test. For example, in the evening we might use levofloxacin, [a broad spectrum antibiotic] (2-1-20180131).

While informants emphasized patient safety as a rationale for prescribing in situations of clinical uncertainty, antibiotics are regarded as quick and effective by both patients and doctors. Furthermore, informants worry that they will lose their clientele if they do not prescribe, or if they are more cautious in prescribing antibiotics than other local doctors.

However, it needs to be emphasized that these uncertainties resulting in greater antibiotic use are not unique to health workers in rural China. For example, among physicians in Australia, Broom et al. (2014, p. 84) identified that all of their informants reported to prefer to err on the side of overuse of antibiotics to reduce immediate risks to patients. “Overtreatment utilizing broad-spectrum [antibiotics], prescribing prophylactic antibiotics, or beginning antibiotics without a clear rationale was viewed as situated within a sense of risk that overtreatment was more favorable than the potential for adverse immediate patient outcomes. In many respects this was about peer perspective and reputational risk.” Furthermore, distinguishing between types of infection has been identified as a challenge common to primary care clinicians^5^ across many settings including the UK, Australia, and North America (Lock and Nguyen, 2010; Cabral et al., 2015; Podolsky, 2015; Broom et al., 2020). Such ambiguities problematize the popular construction of a pure biomedicine that is globally practiced in a hegemonic manner and that somehow exists outside of time, place, and social influence.

## Unpacking Social Drivers of Antibiotic Prescribing

The previous section describes when and how antibiotics and related technologies are employed by informants and how these practices and decisions are understood and rationalized by practitioners. This section considers the wider social and institutional contexts for these decisions among informants from both THCs and VCs and interprets our findings with reference to Bourdieu's theory of capital.

### The Cultural, Economic, and Social Capital of Practitioners

Simply put, cultural capital is the social distinction acquired through the embodiment of social values and assets, as reflected in what one knows, how that knowledge was acquired, and the extent to which social values, skills, knowledge, and taste are embodied (Pinxten and Lievens, 2014). Whereas, social capital can be understood as the resources and social status acquired via one's social relationships, as exemplified in the proverbial “Old Boy's Club.” Social capital is particularly central to Chinese social relations, where success and status are portrayed as a direct outcome of the strength of one's connections or *guanxi* (關係). The concept of *guanxi*, which dates to Confucian doctrine, is enacted through one's mutual commitments and reciprocity in social relations, and is similar to the transcultural role of gift giving—as the crossing of a threshold marking entry into social relationships—as described by Mauss (2002). Social hierarchy is also determined by material assets that are convertible into money, or economic capital. Hence, cultural and economic capital are closely linked with the social capital of *guanxi*, and these three forms of capital intersect and mutually determine one another.

The structure of healthcare in China reflects an embedded hierarchy of cultural and social capital in which the rural VC and its village doctors are positioned at the bottom of this hierarchy, with THCs considered only one step above. Public trust in higher-level health facilities is a reflection of the assignment of cultural capital to the better-qualified staff working in the upper strata of the healthcare system by both the lay public and health workers. Informants from both VCs and THCs denigrated their own lower-tier health facilities by referring to working “down below here,” “down here in the village,” and “at the bottom level.” One of the female VC informants with over 20 years of medical experience stated: “I feel like the frog at the bottom of the well.” She and her colleagues considered themselves to have markedly little influence and importance from their position as workers in lower-tier health facilities. Several other informants mentioned the lack of opportunities for further professional training to improve their qualifications, pointing to their limited medical experience and limited expertise in the health system. In this instance, limited cultural capital not only restricts the capacity to influence others, but also limits access to the means to increase one's cultural capital.

Social capital is also relevant to antibiotic prescribing practices because of its potential impact on clinical knowledge, uncertainty, and treatment decision-making. Village doctors reported that they face greater clinical uncertainty—and thereby need to prescribe more antibiotics in order to reduce patient risk—than their counterparts in higher-tier hospitals. When asked what could be done to tackle antimicrobial resistance, one THC informant spoke about rural clinicians' low “social status” (*shehui diwei*, 社会地位):

The social status of the base-level doctors is low. You are in contact with people who are suffering from bitterness and difficulties and who usually do not have [*sic*.] a good mood. Communication with them can easily lead to medical disputes. If the medical treatment works to their recovery, your job is done, but if it doesn't work, you will be blamed (2-1-20180115).

Thus, for village doctors, prescribing antibiotics may serve to cope with insecurities they experience due to their position at the bottom of the healthcare hierarchy. However, social rank not only is based on a health worker's education, professional status, or career development, but also extends to, and derives from, their access to material resources. For example, in addition to a lack of access to newer-generation antibiotics, informants stated that the medicines available at the “lower-tier facility” were characterized as “medicine of low taste, low grade, inadequate” and “not worth much money” (*bu zhiqian*, 不值钱) (2-1-20180115). Informants commonly differentiated between rural health facilities and upper-level hospitals beyond THCs, which were considered “more advanced” (*gaoji*, 高级), and stocked with “newer antibiotics” (2-1-20180115). Hence, in this instance, we can see the intersection of social, cultural, and economic capital.

Medical materials were not limited to pharmaceuticals but also include access to testing equipment. The example of one village doctor who commented that she could afford to set up blood testing equipment at her clinic demonstrates the mutually determining character of different forms of capital. This doctor commented that offering blood tests could have generated extra revenue for the clinic, but she decided against the purchase, “because people would trust the test results from the township heath center more [than from my village clinic]” (1-3-31). Instead, she commonly sends patients with a fever to the THC for a blood test.

Furthermore, the generation of economic capital was also a factor impacting treatment decisions and the extensive use of medical materials. The 2009 National Essential Medicines Scheme (NEMS) prohibited any increase above the retail price paid to the manufacturer of essential medicines, and was implemented to counteract the long-standing problem of marking up the prices of medicines (procured wholesale) for retail sale to patients in order to generate profit for health facilities and their staff. VC and THC doctors in Anhui province can only prescribe medicines from the *Anhui Provincial List of Essential Medicines*, and a general concern voiced by informants was that the antibiotics on the list are older-generation drugs that are no longer effective. This policy change resulted in a significant loss of income for village and township doctors and has generated its own perverse economic incentives. To make up for the loss in revenues from mark-ups on pharmaceuticals, doctors have found other means to generate income, including prescribing unnecessary diagnostic tests.

These diagnostic tests were understood by informants as essential to their professional survival. For example, one THC director spoke of his ambition to purchase more test equipment to be housed in his new health center building: “the money made from carrying out tests for patients is 100% income, as the investment in the test machine is a one-off.” During site observation, we noted that ~30% of RTI patients with common symptoms were given tests, such as blood tests and X-rays, as presented in Table 2. Thus, although doctors emphasized the use of these tests for guiding antibiotic prescribing in our interviews, neither of these tests reliably indicate the presence of bacterial infection, though economic incentivization may be another, possibly more significant, driver. Given the pressures placed on health workers to generate revenue for their health facilities, doctors are incentivized to order diagnostic tests irrespective of clinical need.

**Table 2:** Tests ordered for the 638 patients observed at THCs.

**Types of test**	**Blood test**	**X-ray**
Carried out	160	107
Not carried out	13	6
Subtotal	173	113

The same issues may help to account for the pervasive use of intravenous administration of drugs. One doctor (2-1-20180115) mentioned that generating revenue for his THC was one of three major pressures for him to prescribe the use of intravenous drips, albeit often using TCM formulae, which are not part of the Essential Medicine List, and thereby can be “marked up.” He describes this practice as his compromise both for patients who do not require any antibiotics but who demand “drip” treatment and for his hospital demanding increased revenues. The same compromise was mentioned by other informants for patients who demand antibiotics and are provided to them, irrespective of their diagnosis.

The ambiguous employment status of VC doctors who are not directly state-salaried, the relatively meager basic salary of THC doctors and the pressures placed on them to generate income for their health facility, and the complex and sometimes lengthy process for obtaining medical insurance reimbursement, all act as direct or indirect economic drivers in treatment decisions^6^.

Regardless of conflicting social pressures placed on health workers, the bottom line for many informants is practicing in a manner that ensures their economic survival. Clearly, the social, cultural, and economic capital of the practitioner interact in complex ways to influence antibiotic prescribing practices. However, the social and cultural capital *of patients* also influence prescribing practices. By refusing patient expectations and demands, informants identify that they risk being overlooked in the local healthcare provider market, which they perceive as the fault of their patients' “low human quality” and poor medical literacy.

### The Cultural and Social Capital of Patients

Doctors' prescribing behaviors are not only related to their own cultural and social capital, but are influenced by their perceptions of their patients to be lacking cultural capital. In general, informants described patients as having “*di suzhi,”* literally low or poor human quality. *Suzhi'* (素质) is a popular Chinese concept that groups people into hierarchies according to their level of self-cultivation, education, and personal achievement, which is embodied in their behavior. Informants referred to patients with *di suzhi* in derogatory terms and emphasized the difficulty in refusing such patients' demands for antibiotics.

One informant offered:

Here in the countryside [patients] are not as civilized (*wenmin*, 文明) as those in the cities. They just come in to demand strong or good water [IV drips] and what can you say to these rough people? (1-3-31).

Several other informants made similar comments, emphasizing their patients' lack of civil behavior or medical knowledge due to residing in a rural area. For example, one informant remarked on when he would refuse patients' requests for antibiotics:

Those with wider knowledge and more education know about [drug resistance], and I will mostly explain [my rationale] to them. But there are some difficult people; they simply come in to say they want antibiotics. I'll explain at most twice [before giving what they ask for]. What if he [the patient] has a bacterial infection? It will backfire on me. Some villagers are particularly stupid and stubborn (2-2-21).

Referring to patients who come in to ask specifically for antibiotics (*xiaoyan yao*, 消炎药), another informant stated:

This is about culture. They are not very highly cultured. Very culturally refined people would not do this [i.e., demand antibiotics]. Rural people believe a more expensive drug is a good drug. People in the countryside think that a higher price is equated with a better drug. More accomplished people, people with more self-cultivation, don't want to take a lot of anti-inflammatories (2-2-201802).

Again, rural patients are portrayed here as lacking cultural capital and as ignorant of the potential consequences of taking many antibiotics. This familiar scenario of blaming the patient for being an ignorant “other” is a form of alterity that has been afforded to the privileged and those proclaiming expertise across many periods and contexts, but in this case, it frees the health worker from accountability or the need to take responsibility for their own prescribing practices. Here, clinicians are using their patients' lack of cultural capital as an excuse for frequent prescribing of antibiotics.

Language is also an important indicator of cultural capital that was emphasized by informants. The terminology for antibiotics used in patient–clinician encounters is an important influence on patients' understanding and use of antibiotics, as we describe elsewhere (Lambert et al., 2019). In consulting with patients, a majority of informants were observed to use the term *xiaoyan yao* (literally translated as anti-inflammation medicine) to refer to antibiotics, such as amoxicillin and cephalosporin. When queried, one clinician explained:

The commoners won't understand if I say *kangsheng su* (抗生素), the technical term for antibiotic. They do not know the term *kangsheng su*. They only know *xiaoyan yao*. These rural folks talk about *xiaoyan yao* out of habit (1-2-15).

Similarly, an in-house pharmacist commented:

*Xiaoyan yao* is local dialect (lit. ‘earthy language’). Between clinicians, we cannot say *xiaoyan yao*. We need to speak formally. In fact, [the patients] use it to mean antibiotics. But if I say “*kangsheng su*,” the patient won't understand. So we need to speak a simple language with them, and discuss *xiaoyan yao*. But if we talk with other doctors using the term *xiaoyan yao*, we would be laughed at (2-2-180131).

A village doctor echoes that he generally would use the term *kangsheng su*, rather than *xiaoyan yao*, with people whose educational level is junior college or higher, whereas “It's pointless to talk about it with ordinary commoners” (2-2-201802). Through this narrative of the “ignorant patient,” doctors perpetuate misunderstandings of the drug and its inappropriate use by using the vernacular term with patients, thereby thwarting the possibility for altering the patient's understanding. This is particularly problematic in China, where patients commonly purchase antibiotics in retail pharmacies without a prescription by seeking the more general category of anti-inflammatories (*xiaoyan yao*).

Patients are *othered* by doctors not only according to their lack of cultural capital (concerning what they don't know), but also according to the cultural and social capital derived from where they live and who they know. Some informants suggest that exposure to the city seems to increase people's “self-cultivation” and medical knowledge:

Young people are better if they have been working as migrants in the city. Otherwise, [there is] no point explaining drug resistance to them, they won't understand (2-2-21).

The implication here is that patients have different inherent qualities based on where they live, though every patient is obliged to take responsibility for their own well-being. By characterizing patients this way, informants, again, appear to shift responsibility for antibiotic prescribing decisions away from themselves and onto the patient.

Rural migrant workers, or the so-called *nongmin gong* (农民工), are commonly portrayed as peasants of *di suzhi* (low human quality) who carry disease and have often been blamed by both the lay public and the medical community in China for the spread of diseases such as HIV/AIDS and tuberculosis into urban areas. This narrative is perpetuated even though the majority of migrant workers are young and healthy and despite the fact that the epidemiological evidence clearly contradicts this narrative (Hesketh et al., 2006; Wang et al., 2007; Gong et al., 2012; Mou et al., 2013). Informants portrayed migrant workers as adding to their difficulty in making treatment decisions. For example, one doctor discussed how massive rural-to-urban migration affects his work:

Many families here are from “left-behind” households, with young parents working as migrants away in the city [while grandparents look after the preschoolers or school-age children]. Few of them demonstrate filial piety (*xiao*, 孝) [being supportive of elderly parents' healthcare] (2-1-20180115).

This doctor went on to discuss how young adult siblings fight over who should pay the medical fees of their elderly parents' and how clinicians often become an easy target for blame in such disputes. Other informants spoke of bearing the brunt if any social problems arise, claiming that rural doctors serve as scapegoats, literally “cannon fodder” (*dang pao hui*, 当炮灰) (2-1-20180115).

Yet, despite their disparagement of patients' low cultural capital, rural doctors are highly dependent on their patients' social capital to maintain their own livelihoods. Perceived or actual poor care would potentially result in significant loss of clientele through reputational damage in rural communities with strong social networks. Thus, in order to avoid patient disputes and “medical trouble” (*yi'nao*, 医闹), health workers feel obliged to do whatever is necessary to appease the patient and their family, particularly to avoid the cost of financial compensation to patients to settle legal medical disputes. Village doctors are particularly vulnerable to such legal action since they are not fully incorporated into the national healthcare system and essentially operate as licensed private practitioners. Private practitioners are more exposed to risks of litigation in this system and are liable to pay any compensation to patients out of their own pockets if there are any medical accidents. One village doctor (1-3-31) claimed to take notes of all her patients' allergies in her record book and to be able to remember almost all by heart, despite not keeping formal patient records other than recording the medicines prescribed to new patients. Similarly, by prescribing antibiotics, clinicians claim they are demonstrating their concern for their patient's health and the need to protect them from potential further complications, even though they are simultaneously protecting their own livelihoods and economic capital, as well as maintaining their own local social capital.

## Conclusion

This research has examined the clinical, social, and economic influences on antibiotic prescribing that clinicians face every day while working at the lowest levels of health facilities in rural Anhui Province, China. A marked gap has been identified between informants' understanding of what antibiotics are for and how, in practice, they are prescribed on a daily basis. Uncertainty in diagnosis and treatment, due to either lack of knowledge or of material resources, and the need to engage in “safe practice” both result from, and are a potential threat to, informants' cultural, social, and economic capital. Health workers' perception and practice of safety is often contradictory and requires a contorted logic in an attempt to fulfill the conflicting demands of capital. In addition to interacting in a complex and unpredictable environment, these drivers of clinical practice are dynamic and changing, particularly in the Chinese context where healthcare policies may markedly change every few years.

Our study clearly has limitations, in part due to the sensitive nature of this research in China at this time. For example, probing financial incentives as a driver for informant prescribing is difficult, particularly since a number of official policies have been launched to reduce the potential of personal and institutional financial gains from medical treatment. We are also aware that informants' characterizations of the ignorance of their patients may be influenced by the interviewing situation, in which practitioners who are being questioned about their clinical practices may, in defense, emphasize their own knowledge to justify their prescribing decisions. Sample size also restricts the representativeness of these data and opportunities to conduct extended ethnographic research beyond the clinic settings were restricted due, again, to sensitivities regarding the conduct of this research in rural areas. Overall, identifying how the overuse of antibiotics and the increasing risk of AMR may best be mitigated in the context of medical institutional hierarchies will benefit from further empirical research. Despite these limitations, we provide new insights into the powerful sociocultural, economic, and institutional drivers that shape antibiotic prescribing, in addition to a fresh analytic approach that demonstrates that prescribing practices cannot be explained solely by reference to individually modifiable forms of behavior.

Our work largely corroborates, but goes beyond, the key findings of Broom et al. (2014) in which maintaining forms of capital often outweighed adherence to clinical guidelines, including antimicrobial stewardship, in the very different setting of Australian hospitals. Although our findings align with the conclusion that clinicians are placed “in the complex position of abiding by obligations and responsibilities (to patients, peers, institutional structures, profit, recovery) while also navigating the values of scientific rigor and efficacy (a complex balance of social capital (networks), cultural capital (reputation), and professional capital)” (Broom et al., 2017, p. 2003), previous research has largely focused only on the influence of social capital. In this paper, we have attempted to further develop the use of Bourdieu's concept of capital to examine clinician antibiotic prescribing practices by also considering cultural and economic forms of capital as particularly salient in this setting and thereby offer a valuable and original perspective for better understanding the sociocultural factors impacting AMR.

## Data Availability Statement

The raw data supporting the conclusions of this article will be made available by the authors, without undue reservation.

## Ethics Statement

This study involving human participants was reviewed and approved by Anhui Medical University Ethics Committee. Participants provided their written informed consent to participate in this study.

## Author Contributions

HL, CC, PK, and MC planned and designed the interview topic guides. MC conducted interviews in all field sites, coded all interview transcripts, translated the quotes, and drafted the first manuscript version. PK contributed the theoretical framework. All authors contributed to the data analysis and reviewed and revised the final version of the manuscript.

## Conflict of Interest

The authors declare that the research was conducted in the absence of any commercial or financial relationships that could be construed as a potential conflict of interest.

## Footnotes

^1^*Habitus* is Bourdieu's conception of the physical embodiment of social and cultural capital manifested through physical actions and activities.

^2^For a full discussion of the forms of capital, see: Bourdieu (1986) *The Forms of Capital*.

^3^For further details of China's primary health care system, see, for example, Li et al. (2017) and Duckett (2012).

^4^For example, village doctors are not included as health professionals in formal Chinese health statistics (Hu et al., 2017:7).

^5^This lack of differentiation is equally problematic among the lay public in the west. For example, Lock and Nguyen (2010:4-5) identify that though “Canadians believe that antibiotics are effective against viruses, two-thirds of Canadians report that they have a clear understanding of when and how antibiotics should be taken.”

^6^The vast majority (over 99%) of villagers in all three of our sites were members of the NACMIS (New Agricultural Cooperative Medical Insurance Scheme, *xin nong he*, 新农合). VCs and THCs have to work with NRCMIS, but the process of insurance reimbursement for a patient's medical care is not straightforward and cannot be covered here (see Bernardi and Miani, 2014).
